# Association between Empathy and Clinical Symptoms among Overweight and Non-Overweight Chinese Chronic Schizophrenia Patients

**DOI:** 10.3390/brainsci13071075

**Published:** 2023-07-15

**Authors:** Yuchen Li, Ruichenxi Luo, Dongmei Wang, Xiangyang Zhang

**Affiliations:** 1 School of Mental Health and Psychological Sciences, Anhui Medical University, Hefei 230032, China; liyuchen1001@163.com (Y.L.); lrcx5729@163.com (R.L.); 2 Institute of Psychology, Chinese Academy of Sciences, Beijing 100101, China; wangdm@psych.ac.cn; 3 Department of Psychology, University of Chinese Academy of Sciences, Beijing 100101, China

**Keywords:** schizophrenia, empathy, clinical symptoms, overweight

## Abstract

Patients with schizophrenia are afflicted by severe clinical symptoms and serious cognitive dysfunction. The aim of this study is to investigate the potential relationships between clinical symptoms and empathy and their variations between overweight and non-overweight schizophrenia patients. To address this problem, a group of 776 inpatients diagnosed with chronic schizophrenia (504 overweight patients and 272 non-overweight patients) was recruited. The Positive and Negative Syndrome Scale (PANSS) and its five-factor model were employed to assess clinical symptoms, while empathy levels were measured using the Interpersonal Reactivity Index (IRI). The overweight patients had lower education levels but higher positive symptoms than the non-overweight patients (all *p* < 0.05). In addition, the overweight patients performed significantly better with respect to empathy (FDR-corrected *p* < 0.05). Additional multiple regression analyses indicated significant associations between the total score of the IRI and PANSS negative symptoms, gender, and family history of psychiatric disorders among the overweight group; among non-overweight patients, there was a significant correlation between suicide and the total score of the IRI. This study provides evidence suggesting that chronic schizophrenia patients who are overweight may have distinct clinical characteristics, particularly with respect to their empathy, compared with non-overweight patients. Moreover, different variables are associated with empathy in different groups.

## 1. Introduction

Schizophrenia is one of the most common serious mental illnesses and numbers among the most serious disabling diseases [1]. With the discovery of antipsychotic drugs, the symptoms of schizophrenia have been effectively improved, especially hallucinations and delusions, among other positive symptoms. Although the use of antipsychotic drugs has greatly mitigated the clinical symptoms of schizophrenia patients, most patients experience weight gain and even an increased risk of developing metabolic syndrome [2,3,4]. Epidemiological studies have shown that the rate of overweight and obesity among Chinese schizophrenia patients is 44.8% [5], which is significantly higher than that among healthy control group [6]. In addition, numerous studies have consistently found that individuals with schizophrenia are 2–4 times more likely to develop metabolic syndrome than the general population [7,8].

There are various causes of weight gain among schizophrenic patients; for example, second-generation antipsychotics can lead to increased food intake, binge eating, and energy expenditure, which interfere with a patient’s eating behavior [9,10]. This may be related to a patient’s leptin levels [11] and affinity for histamine H1 receptors [12,13], D2 receptors, and 5-HT2C receptors [14]. Experimental evidence suggests that olanzapine increases an individual’s preference for high-fat/high-sugar diets [15,16]. In addition, a large number of factors are related to the enhanced risk of obesity among schizophrenic patients, such as being female, older, less educated, sedentary, lacking with respect to exercise [17], and having poor dietary choices [18]. Also, studies have shown that increased weight among patients leads to higher rates of metabolic diseases, for instance, cardiovascular disease and diabetes [19,20], which increase mortality [21,22]. Consequently, patients with schizophrenia have a life expectancy of 10 to 15 years less than their peers without psychiatric disorders [23,24]. Various studies have reported that individuals who are obese tend to exhibit lower self-esteem [25], higher rates of depression, and an elevated risk of suicide [26,27]. Therefore, weight gain is a key issue for patients with schizophrenia and needs to be taken seriously.

A large number of studies have demonstrated an association between increased weight and clinical symptoms among individuals with schizophrenia [5,28,29,30]. A study involving Chinese patients with chronic schizophrenia found a positive association between improvement in psychopathological symptoms (like negative symptoms) and obesity [29]. In another study of first-episode schizophrenia patients, it was shown that increases in PANSS positive symptoms, general psychopathological symptoms, and total scores were significantly associated with weight gain [5]. One of the major characteristics of schizophrenia, neurocognitive impairment, has been highlighted by numerous researchers [31,32]. According to numerous studies, schizophrenia patients exhibit cognitive impairments across multiple domains, such as information-processing speed, attention/alertness, working memory, verbal learning, and social cognition [33,34,35,36], suggesting that neurocognitive function remains a major focus of studies on schizophrenia. In comparison, less attention is paid to social cognition, which is important for the prognosis of patients’ social and personal daily functioning. Several studies have indicated that schizophrenic patients also present social cognitive impairments, mainly in the form of difficulties with emotion recognition and understanding the thoughts of others as well as treating others emotionally [37,38]. A meta-analysis showed that social cognitive functioning was more strongly associated with outcomes of functioning among schizophrenia patients compared to neurocognition [39].

Empathy, as an important social cognitive function, involves sharing, understanding, and responding to the emotional experiences of others and usually includes both cognitive and affective components, suggesting that cognitive empathy refers to the cognitive process of comprehending and recognizing the emotions of others, while affective empathy relates to the emotional experience of sharing in the feelings of others [40]. However, research has found inconsistent results regarding cognitive and affective empathy among schizophrenia patients. Many reports have found deficits in cognitive empathy among patients with schizophrenia, but there has been controversy regarding affective empathy. For example, according to a meta-analysis, people with schizophrenia have severe impairments in emotional empathy [41], suggesting that schizophrenic patients have a reduced ability to experience emotions. Nonetheless, some studies have indicated that patients with schizophrenia do not exhibit significant differences in affective empathy when compared with that of healthy individuals [42,43]. Several studies even suggest that people with schizophrenia can exhibit a greater degree of affective empathy [44,45]. Thus, there is no consensus on the research regarding empathy among schizophrenia patients. In addition, it is unclear whether obese schizophrenics have impairments in empathy. A recent study found that Theory of Mind (TOM) may correlate with metabolic dysfunction among males suffering from schizophrenia, showing that BMI has an impact on both affective and cognitive theory of mind among male patients [46]. However, a study with inconsistent findings on the association between GNAS (guanine nucleotide binding protein, alpha stimulating) gene polymorphisms and cognitive empathy in healthy female populations reported that C393C allele carriers had higher Interpersonal Reactivity Index (IRI) perspective-taking (PT) scores in comparison to T allele carriers. Furthermore, it is noteworthy that individuals with the C393C allele have been found to have an elevated risk of metabolic disorders, including hypertension, obesity, and diabetes [47].

Many studies have confirmed the connection between clinical symptoms and empathy, which suggests that clinical symptoms are predictors of empathy among schizophrenia patients. For instance, one study discovered statistically significant relationships between empathy and negative symptoms and general psychopathology among chronic schizophrenic patients [48]. In addition, numerous previous reports revealed that empathy was markedly related to negative symptoms among schizophrenia patients in both subjective reports and based on behavioral assessments [49,50,51]. However, the link between positive symptoms and empathy is uncertain, as some studies have shown no relationship between them [52], whereas several studies have found a weak correlation between positive symptoms and empathy [48]. Furthermore, there is a significant association between anxiety, depression, cognitive impairment, and empathy among patients with schizophrenia [48,53,54]. Thus, no definite conclusions have been drawn regarding this issue. 

Based on our current understanding, there is no existing research that has focused on exploring the correlation between empathy and overweight status among patients who have been diagnosed with schizophrenia. Therefore, this relationship deserves to be investigated intensively in the Chinese population. Such an investigation may help to improve our understanding of the treatment of specific patient groups. The main purpose of the current study is to investigate the potential differences in empathy and clinical presentation between patients with schizophrenia who are overweight and those who are not by using a cross-sectional design. This study had two main objectives: (1) to determine empathic ability assessed via IRI among Chinese overweight versus non-overweight schizophrenic patients and (2) to reveal the association between empathy and clinical symptoms in both patient groups. We proposed the following hypotheses. (1) Empathic abilities will be better in the overweight group than in the non-overweight group. (2) Clinical symptoms, especially negative symptoms, will correlate significantly with empathic ability.

## 2. Materials and Methods

### 2.1. Participants

From January to April 2019, 776 inpatients (male/female = 514/262) were recruited from four psychiatric hospitals located in Wuhan and Guangzhou. Trained investigators obtained general information, sociodemographic data, medical history, and the results of physical examinations and laboratory tests for all participants using a self-designed questionnaire.

The inclusion criteria and steps through which they were enforced were as follows: (1) patients had to be Han Chinese, aged between 18 and 70 years; (2) to ensure the accuracy of the diagnosis of schizophrenia, two trained psychiatrists independently utilized the Structured Clinical Interview of the DSM-IV (SCID) for diagnostic purposes; (3) patients’ duration of illness had to be at least one year; and (4) all patients had to have been receiving a consistent dose of antipsychotic medication for a minimum of 6 months prior to enrollment in the study. In addition, exclusion criteria were as follows: (1) comorbid serious physical illness (e.g., cardiovascular disease, infectious disease, cancer, and immune system disorders); (2) combination of severe neurological disorders or intellectual disability; (3) significant psychiatric symptom fluctuations in the last 2 weeks; and (4) pregnant or lactating women.

We obtained approval for the study protocol from the Institutional Review Board of the Institute of Psychology, Chinese Academy of Sciences. Prior to participation in this study, all participants or their legal guardians provided written informed consent.

### 2.2. Clinical Assessment

Six psychiatrists with more than 5 years of experience in clinical practice used the Positive and Negative Syndrome Scale (PANSS) to evaluate the psychopathological symptoms of the participants [55]. To ensure consistency and reliability, prior to initiating the study, the 6 psychiatrists attended a training course on using the PANSS. Thereafter, in a repeated assessment, they maintained an intra-rater correlation coefficient of 0.85 for the PANSS total score.

Traditionally, the PANSS is composed of positive, negative, and general psychopathology symptoms. However, we employed a novel five-factor model of the PANSS based on recent findings [56,57,58]. The five distinct components of the PANSS five-factor model are as follows: “Positive”, “Negative”, “Cognitive”, “Depressive”, and “Excitement”.

### 2.3. Empathy

In this study, the Interpersonal Reactivity Index (IRI), consisting of 28 items, was utilized to assess patient empathy [59]. The IRI has been frequently employed to gauge empathy among patients diagnosed with schizophrenia. It comprises four domains: Perspective Taking (PT), Fantasy (FS), Empathic Concern (EC), and Personal Distress (PD). The Cronbach’s α coefficient for the IRI was 0.775, and the Cronbach’s α coefficients for the factors ranged from 0.481 to 0.631. Although the Cronbach’s α coefficients for the factors were low, they were still at an acceptable level. This indicates that the scale has good internal consistency and good reliability. For the confirmatory factor analysis of scale, the four-factor model fit indices were χ^2^/df = 2.459, RMSEA = 0.043, GFI = 0.922, and AGFI = 0.906. The fit indices all reached a satisfactory level and had good validity.

PT is an individual’s tendency to spontaneously adopt the perspective of others and most clearly represents the cognitive aspect of empathy. FS is the ability to imagine and empathize with characters in virtual environments such as books, films, and television programs. EC most obviously corresponds to emotional empathy and refers to a tendency to empathize with and show concern for someone in a miserable situation. The PD scale measures an individual’s level of anxiety and discontent when they are in a stressful interpersonal environment. PT and FS are typically regarded as measures of cognitive empathy, while EC and PD are indicators of affective empathy. Previous studies conducted on Chinese populations have shown good clinical efficacy and test–repeat reliability of the Chinese version of the IRI [60].

### 2.4. Measurement of Anthropometric Variable

Standardized procedures were followed to measure weight and height of participants, and based on these measurements, we calculated the patients’ body mass index (BMI) values (kg/m^2^). The height of the participants was measured with an accuracy of one millimeter when standing upright and barefoot. Participants were instructed to wear light clothing and their weight was measured using an electronic scale with a precision of ±0.1 kg. We classified patients as either non-overweight (<23 kg/m^2^) or overweight (≥23 kg/m^2^) according to the standard BMI classification for Asians provided by the World Health Organization [61]. A BMI greater than or equal to 28 kg/m^2^ indicates that a person is obese [62].

### 2.5. Statistical Analysis

We used the χ^2^ test and the independent samples *t*-test to compare differences in demographic and clinical variables. Subsequently, correlations between clinical variables and empathy were analyzed using Pearson’s or Spearman’s correlation, as appropriate. Multivariate stepwise regression analysis, using the dependent variable of IRI total score, was further conducted to explore the variables related to empathy in the overweight and non-overweight groups. 

We adjusted for multiple testing using the false discovery rate (FDR) method and conducted all statistical analyses using SPSS version 24.0. Statistical significance was determined as a two-tailed *p*-value < 0.05.

## 3. Results

### 3.1. Prevalence-Related, Clinical, and Demographic Characteristics of Overweight Patients

The demographic characteristics of all patients who were included in this study are presented in Table 1. The prevalence of overweight/obesity among patients with chronic schizophrenia was 64.9%, which was higher than that of the healthy southern Chinese population (33.7%) [6] and that reported in another epidemiological study of obesity in China (50.9%) [63] and similar to values reported in previous studies (40–70%) [5,64,65,66]. Also, the prevalence of obesity among patients with chronic schizophrenia was 18.9%, and there was no statistical difference in overweight/obesity rates between the male (62.8%) and female (69.1%) patient groups.

Compared to the non-overweight patients, overweight patients had fewer years of education (*p* = 0.001, FDR corrected *p* < 0.05), higher suicide rates (*p* = 0.015), and higher PANSS positive symptoms scores (*p* = 0.014; FDR corrected *p* = 0.056) but lower PANSS negative symptom scores (*p* = 0.025; FDR-corrected *p* = 0.061). (Table 2).

### 3.2. Comparison of IRI Scores between Non-Overweight and Overweight Patients

All patients completed the IRI. Table 3 shows the IRI scores of the participants. Compared to the non-overweight patients, the overweight patients performed better with respect to the IRI total score and PT and EC scores (all FDR-corrected *p* < 0.05).

### 3.3. Relationship between Overweight and Empathy

Among all participants, the IRI total score showed negative correlations with age, age at first hospitalization, PANSS negative symptoms, general psychopathology, and total score but positive relations to education (all FDR-corrected; *p* < 0.05). Further multiple regression analysis using BMI as the dependent variable showed that IRI-based perspective taking (β = −0.071, t = 1.977, *p* = 0.048, and R^2^ change = 0.005), PANSS positive symptoms (β = 0.130, t = 3.591, *p* < 0.001, and R^2^ change = 0.016), PANSS negative symptoms (β = −0.077, t = −2.174, *p* = 0.040, and R^2^ change = 0.009), education years (β = −0.125, t = −3.469, *p* = 0.001, and R^2^ change = 0.10), and age (β = −0.077, t = −2.174, *p* = 0.030, and R^2^ change = 0.006) were associated with BMI.

Table 4 and Table 5 present the correlation coefficient matrices for empathy and clinical symptoms among the non-overweight and overweight patients, respectively. In the overweight group, PT, EC, and IRI total scores were negatively related to PANSS negative symptoms, general psychopathology, and total scores (FDR-corrected; all *p* < 0.05). However, no remarkable relationships between IRI total score and PANSS scores were observed among the non-overweight patients. In addition, PD was positively correlated with PANSS negative symptoms, general psychopathology, and total scores among the non-overweight patients (FDR-corrected; all *p* < 0.05).

After conducting an additional regression analysis on the overweight patients, we found an independent association between the IRI total score and PANSS negative symptoms (β = −0.202, t = −4.617, *p* < 0.001, and R^2^ change = 0.38), gender (β = 0.094, t = 2.136, *p* = 0.033, and R^2^ change = 0.009), and family history (β = 0.105, t = 2.386, *p* = 0.017, and R^2^ change = 0.013). In addition, among non-overweight patients, suicide was only associated with IRI total score (β = 0.171, t = 2.834, *p* = 0.005, and R^2^ change = 0.029).

## 4. Discussion

To the best of our knowledge, our study appears to be the first study conducted in China that investigates potential associations between overweight status, empathy, and clinical symptoms among schizophrenia patients. Some of the principal discoveries of this study were as follows: (1) The overweight group showed greater empathy than the non-overweight group. (2) Among the overweight patients, PANSS negative symptoms, gender, and family history were independently associated with the total score of the IRI. (3) Among the non-overweight patients, suicide was only associated with IRI total score.

This study revealed that overweight patients exhibited higher levels of empathy compared to non-overweight patients. Although there is a paucity of literature on the empathic capacity of overweight patients, our finding is consistent with that of a previous study reporting that a metabolic-dysfunction-related gene was associated with higher cognitive empathy among female participants [47]. Moreover, another study found that among females with schizophrenia, those with higher BMI scores possessed a greater capacity for empathy [67]. We hypothesized that there are several explanations for these findings. First, our study observed that overweight patients scored lower in terms of negative symptoms but higher in terms of depressive symptoms according to the PANSS five-factor model. One study investigated the link between negative symptoms and empathy among individuals diagnosed with schizophrenia; the authors consistently found associations between them [48], suggesting that the fewer negative symptoms a patient has, the greater their overall ability to empathize. Furthermore, studies have shown that greater depressive symptoms are associated with greater emotional empathy [48,68], which might explain the greater empathy among overweight patients with greater depressive symptoms. Second, oxytocin (OXT) has been extensively studied in terms of its being a key factor linking social signals to cognition, behavior, and reward [69]. OXT is central to the regulation of many social–behavioral domains, like emotion perception and empathy [70], and it also promotes the neuronal growth of specific brain regions related to social behavior and positive emotion induction [71]. Also, this study found that the obese group had significantly higher OXT levels than the healthy group, and these levels were associated with BMI and body fat levels [72]. Furthermore, the higher the serum OXT level, the higher the risk of metabolic syndrome [73]. Studies have demonstrated the use of OXT as a novel drug to treat social–behavioral deficits, such as emotion recognition and empathy, among patients with psychiatric disorders [70,74,75,76], with the results showing significant improvements in these symptoms after treatment. Also, one study revealed that men with schizophrenia who had a significantly lower BMI were worse at recognizing joyful emotions, and it found a significant positive correlation between the recognition of joyful emotions and IRI perspective taking [67]. In one large cohort study, there was an association between a common oxytocin receptor (OXTR) gene polymorphism (rs53576) and emotional empathy [77]. OXT requires the activation of G proteins in order to signal, and a healthy cohort study found a significant correlation between cognitive empathy and the GNAS C393T gene polymorphism involved in the encoding of G proteins. Minoretti et al. [78] hypothesized a possible association between the TT genotype and the schizophrenia deficiency syndrome subtype, for which affected patients typically present poor empathy [79]. However, C393C allele carriers have been shown to have more empathic tendencies, with higher IRI PT scores compared to C393T allele carriers, and the C393C allele has been recognized as a risk factor for several metabolic disorders, including obesity [47]. Third, our study observed a higher suicide rate in the overweight group, and related studies confirm that suicide relates to greater levels of empathy [80]. One study reported that schizophrenics who committed suicide had a higher intensity of von Economo neurons (VEN) located anterior to the cingulate gyrus (which has been widely shown to be strongly associated with empathy) compared to patients with other causes of death [81,82]. Notably, there were more female patients in our overweight group, and an increasing number of studies have shown that females perform better than males in terms of empathy [67,83,84]. However, the empathic difference found in this paper has not been definitively found in healthy populations or among other psychiatric patients, so it would be interesting to validate this result with respect to different people to verify whether this difference is unique to schizophrenics. Overall, the findings of our study suggest that overweight patients with schizophrenia may exhibit better empathy compared to non-overweight patients. As this study is an initial investigation of the association between overweight/obesity and empathy among individuals with schizophrenia, it is necessary to confirm this relationship in further research.

Notably, our results showed that PANSS negative symptoms were lower among the overweight participants, and this result is consistent with previous studies that reported a negative association between BMI and PANSS negative symptoms among chronic schizophrenia patients [29,30]. More interestingly, our study only showed a significant and independent association between negative symptoms and the empathy total score in the overweight group, thus corroborating previous research findings. This strongly suggests that there is a significant link between empathy and negative symptoms among individuals with schizophrenia [48,79]. One study found that lower levels of amygdala activation might be related to negative symptoms and empathy deficits, which could be the underlying mechanisms [85]. In addition, some studies utilizing different empathy assessment tools supported these results based on findings that negative symptoms predicted empathy test results [50,51]. However, some studies did not find an effect of negative symptoms on empathy [86]. Conflicts between previous reports regarding the effect of negative symptoms on empathy likely reflect the influence of schizophrenia subtype groups. In addition, we found a significant contribution of gender to the IRI total score, which is consistent with the previously found significant gender differences regarding empathy [47,67,84]. More interestingly, as in the prior findings, we found a significant association between suicide and IRI total scores among overweight patients [80]. Notably, the lower rate of suicide in the non-overweight group may be explained by the higher levels of negative symptoms, which constitute a protective factor against patient suicide [80,87,88]; additionally, patients with higher negative symptoms have lower suicide rates and are less empathic.

The several limitations of this study should be acknowledged. First, it should be noted that this study’s cross-sectional design restricted our ability to draw conclusions regarding whether clinical symptoms and empathy are directly correlated among patients with both overweight and schizophrenia. Therefore, our findings are only preliminary explorations. Second, our study did not include healthy controls and could neither determine whether schizophrenia patients have impaired empathy nor confirm the degree of impaired empathy among overweight and non-overweight patients. Third, patients with schizophrenia often have a severe cognitive impairment, and the use of the IRI, a self-rated scale widely used to assess empathy, may have the potential to produce relatively inaccurate results. Therefore, in the future, it is advisable to measure empathy in conjunction with other objective assessment tools or measurements in order to arrive at an accurate final result. Fourth, this study did not measure and compare neurocognitive function among overweight and non-overweight schizophrenia patients. Patients with schizophrenia usually suffered from poor neurocognitive function, and it is not known whether being overweight/obese affects cognitive function [89]. Several studies have found a strong link between neurocognitive function, clinical symptoms [90,91], and empathy [79,92]. However, the relationship between them is still inconclusive. Therefore, in further studies, it is necessary to measure and control neurocognitive functions among schizophrenia patients. Finally, the patients recruited for this paper comprised chronic patients with long hospital stays and a concentration of middle-aged patients on long-term medication, all of which contributed to the increased BMI. Therefore, future comparisons incorporating first-episode drug-naïve patients or other different populations are needed to confirm the findings.

## 5. Conclusions

In summary, we revealed that overweight patients showed better empathy than non-overweight schizophrenic patients and that PANSS negative symptoms were found to have a significant independent correlation with IRI total scores among the overweight patients. Furthermore, this study has some methodological limitations, including the use of self-reported measures on the IRI self-reported measures, which might have introduced potential bias in our results; thus, we should interpret these findings with caution and investigate them further in future studies using a longitudinal design to identify additional factors that could explain empathic performance among the overweight schizophrenia patients. In future research, it should also be interesting to explore the differences in empathy across age, education years, race, and ethnicity and investigate whether similar results to this paper can be found in different populations, such as healthy populations and those with other mental illnesses such as depression.

## Figures and Tables

**Table 1:** Demographic and clinical characteristics of schizophrenia patients with or without overweight, N (%) or M (SD).

Index	Total Patients (n = 776)	Overweight (n = 504)	Non-Overweight (n = 272)	χ^2^/t	df	*p*
Gender				2.972	1	0.085
Male	514 (66.2)	323 (62.8)	191 (37.2)			
Female	262 (33.8)	181 (69.1)	81 (30.9)			
Marital status				8.203	3	0.042 *
Single	466 (60.1)	287 (57.1)	179 (66.1)			
Married	173 (22.3)	122 (24.3)	51 (18.8)			
Divorced	123 (15.9)	88 (17.5)	35 (12.9)			
Widowed	12 (1.5)	6 (1.2)	6 (2.2)			
Diabetes				4.249	1	0.039 *
Positive	666 (85.8)	81 (16.1)	29 (10.7)			
Negative	110 (14.2)	423 (83.9)	243 (89.3)			
Suicide history				5.922	1	0.015 *
Positive	680 (87.6)	73 (14.5)	23 (8.5)			
Negative	96 (12.4)	431 (85.5)	254 (91.5)			
Smoking history				2.031	2	0.362
Never smoker	404 (52.1)	265 (55.8)	139 (53.7)			
Former smoker	109 (14.0)	64 (13.5)	45 (17.4)			
Current smoker	221 (28.5)	146 (30.7)	75 (29.0)			
Family history of psychotic disorder				0.006	1	0.940
Positive	658 (84.8)	77 (15.3)	41 (15.1)			
Negative	118 (15.2)	427 (84.7)	231 (84.9)			
Age (years)	46.83 (12.28)	46.51 (11.72)	47.42 (13.25)	0.955	1,774	0.340
Education (years)	9.41 (3.11)	9.13 (3.05)	9.92 (3.17)	3.364	1,774	0.001 **
Age at onset of schizophrenia	25.37 (7.65)	25.49 (7.60)	25.21 (9.84)	−0.429	1,774	0.668
Age of first hospitalization	28.55 (9.96)	28.49 (10.03)	28.65(10.00)	0.207	1,774	0.836
Ill course	21.45 (11.76)	21.05(11.26)	22.21(12.61)	1.271	1,774	0.204
Antipsychotic dose (mg/day) (Chlorpromazine equivalents)	310.71 (230.20)	311.24 (239.67)	309.74 (212.08)	−0.086	1,757	0.929

M: mean; SD: standard deviation. * Significant at *p* < 0.05; ** significant at *p* < 0.01.

**Table 2:** Clinical characteristics of overweight patients and non-overweight patients, presented as M values (SD).

Index	Overweight (n = 504)	Non-Overweight (n = 272)	t	df	*p*
PANSS scores					
Positive symptoms	16.41 (5.62)	15.49 (4.64)	−2.453	1,774	0.014 *
Negative symptoms	20.70 (6.67)	21.50 (6.42)	1.614	1,774	0.107
General psychopathology	39.50 (8.63)	39.51 (8.00)	0.015	1,774	0.988
Total	76.61 (17.40)	76.50 (15.70)	−0.091	1,774	0.928
F1 Positive Symptoms	9.25 (4.15)	8.88 (3.59)	−1.287	1,774	0.199
F2 Negative Symptoms	16.93 (6.28)	17.97 (5.89)	2.251	1,771	0.025 *
F3 Cognitive Symptoms	9.37 (3.11)	9.21 (3.06)	−0.677	1,774	0.498
F4 Depressive factor	7.18 (2.60)	6.77 (2.51)	−2.087	1,772	0.037 *
F5 Excitement factor	7.86 (2.99)	7.33 (2.80)	−2.384	1,774	0.017 *

M: mean; SD: standard deviation; PANSS = Positive and Negative Syndrome Scale. * Significant at *p* < 0.05.

**Table 3:** The empathy of schizophrenia patients with or without deficit syndrome, presented as M values (SD).

Index	Overweight (n = 504)	Non-Overweight (n = 272)	t	df	*p*
Perspective Taking	22.25 (5.13)	21.32 (5.23)	−2.407	1,774	0.016 *
Empathic Concern	22.94 (5.02)	22.07 (5.27)	−2.269	1,774	0.024 *
Fantasy	20.20 (5.63)	19.39 (5.74)	−1.905	1,774	0.057
Personal Distress	20.94 (4.71)	20.90 (4.85)	−0.110	1,774	0.912
IRI total score	86.33 (14.46)	83.67 (15.20)	−2.402	1,774	0.017 *

M: mean; SD: standard deviation; IRI = Interpersonal Reactivity Index. * Significant at *p* < 0.05.

**Table 4:** Matrix of correlation coefficients between empathy and clinical symptoms among overweight patients.

Index	Positive Symptoms	Negative Symptoms	General Psychopathology	PANSS Total
IRI				
Perspective Taking	−0.060	−0.207 ***	−0.186 ***	−0.197 ***
Fantasy	0.062	−0.155 ***	−0.079	−0.071
Empathic Concern	−0.023	−0.147 **	−0.116 **	−0.121 **
Personal Distress	0.005	0.012	0.014	0.003
Total score	−0.005	−0.178 ***	−0.137 **	−0.140 **

** significant at *p* < 0.01; *** significant at *p* < 0.001.

**Table 5:** Matrix of correlation coefficients between empathy and clinical symptoms among non-overweight patients.

Index	Positive Symptoms	Negative Symptoms	General Psychopathology	PANSS Total
IRI				
Perspective Taking	−0.081	−0.152 *	−0.112	−0.143 *
Fantasy	0.033	−0.069	−0.015	−0.026
Empathic Concern	0.007	−0.175 **	−0.075	−0.108
Personal Distress	0.006	0.157 **	0.198 **	0.167 **
Total score	−0.011	−0.089	−0.007	−0.043

* Significant at *p* < 0.05; ** significant at *p* < 0.01.

## Data Availability

The data presented in this study are available from the corresponding author on reasonable request.
